# Detoxification Processes from Vanadate at the Root Apoplasm Activated by Caffeic and Polygalacturonic Acids

**DOI:** 10.1371/journal.pone.0141041

**Published:** 2015-10-20

**Authors:** Giovanni Garau, Amedeo Palma, Gian Paolo Lauro, Elena Mele, Caterina Senette, Bruno Manunza, Salvatore Deiana

**Affiliations:** 1 Dipartimento di Agraria, University of Sassari, Sassari, Italy; 2 Istituto di Scienze delle Produzioni Alimentari, CNR, Sassari, Italy; VIT University, INDIA

## Abstract

In the root apoplasm, V(V) and V(IV) toxicity can be alleviated through redox and complexation reactions involving phenolic substances and the polyuronic components. In such context we report the role of polygalacturonic acid (PGA) on the reducing activity of caffeic acid (CAF) towards V(V). The redox reaction was particularly effective at pH 2.8 leading to the formation of oxidation products with redox activity towards V(V). An *o*-quinone was identified as the first product of the reaction which is further involved in the formation of CAF dimers. At pH ≥ 3.6 the redox activity decreased and a yield in V(IV) equal to 38, 31, 21 and 14% was found at pH 3.6, 4.0. 5.0 and 6.0 respectively compared with that obtained at pH 2.8. The redox reaction was faster in the presence of PGA and a higher yield of V(IV) was found in the 4.0–6.0 pH range with respect to the CAF-V(V) binary system. The higher efficiency of the redox reaction in the presence of PGA was related with the ability of PGA to bind V(IV). The biological significance of the redox reaction between CAF and V(V), as well as the role of PGA in such reaction, was established “in vivo” using triticale plants. Results showed that PGA reduced significantly the phytotoxic effects of the V(V)-CAF system.

## Introduction

Vanadium is nowadays the object of a great attention as environmental pollutant. Its release, due to both natural erosion processes and human activities, such as the widespread use of fossil fuels [1] and phosphate fertilizers, may cause a severe pollution of the soil-water-plant system [2,3].

The mobility and bioavailability of vanadium in the environment is strictly related to its oxidation state, being V(IV) and V(V) the most stable species [4]. In soil, V(IV) is generally precipitated as hydroxide or adsorbed onto mineral surfaces or complexed by organic ligands [5]. Thus, V(IV) has relatively low mobility with respect to V(V) which is more toxic. Recent studies suggest that vanadium is essential for the living systems being an important cofactor in vanadate dependent haloperoxidases and vanadium nitrogenases [6]. Moreover, as phosphate analogue, vanadate can influence the behaviour of several important enzymes such as phosphorylases, mutases, phosphatases, ribonucleases, and ATPases [7]. There are firm evidence that vanadate is absorbed by plant tissues [8,9] and can inhibit the plasma membrane hydrogen (H^+^)-translocation ATPase. Further, V(V) was found to retard the growth of tomato plants and accumulate at higher levels in roots [10]. On the other hand, similar effects were induced by the oxovanadium(IV) cation which drastically altered soybean nutrition and reduced plant biomass when added to hydroponic solutions [11]. Morphological changes of roots and leaves of *Phaseolus vulgaris* were also reported for plants exposed to V(IV) [12].

Caffeic acid (3,4-dihydroxycinnamic acid), is recognized to have an important role in several biochemical processes associated in lowering the risks of cancer and cardiovascular disease being able to act as antioxidant and protect against free radicals and viral infection [13,14]. In addition, caffeic acid and its derivates, play an important role in the uptake of iron by plants through its reduction to Fe(II) [15–18] and on the detoxification processes of heavy metals such as Cu(II) and Cr(VI) through their reduction to Cu (I) and Cr (III) [19–21]. Similarly, CAF might be active in the reduction of V(V).

In order to determine the mechanism through which CAF could operate the reduction of V(V) and to evaluate the possible role of the root polyuronic components in this mechanism, the V(V)-CAF system was studied in the aqueous phase, as a function of time, in the presence and absence of polygalacturonic acid (PGA), in the 3.0–6.0 pH range and at different V(V)/CAF molar ratios.

Polygalacturonic acid, the main component of root apoplasm and root cape slime [22–25], plays an important role in the sorption of micro- and macronutrients by plants being able to bind them through the formation of complexes of different nature [26]. Electrostatic interactions occur for some ions such as Ca(II), Mn(II) and Zn(II) whereas inner-sphere binding through carboxylate groups occurs for ions such as Cu(II) and Pb(II) [27].

To evaluate the potential biological significance of the CAF-V(V) system, as well as the role of PGA on the V(V)-CAF interaction, triticale plants (x *Triticosecale* Wittm.) were grown at pH 6.0 in hydroponic solutions containing V(V), V(V)˗CAF and V(V)˗CAF˗PGA. Measurements of root and shoot length were used to estimate the phytotoxicity of each system.

## Materials and Methods

### Chemicals

All reagents were obtained from Fluka. Sodium metavanadate and vanadyl sulfate were employed as V(V) and V(IV) source, respectively. All solutions were prepared just before the beginning of each experiment using Millipore MilliQ ultra-pure water and brought to the working pH by adding 0.001 N HClO_4_ or NaOH. Sodium perchlorate monohydrate was used as the supporting electrolyte at 0.01M.

### V(IV) and V(V) determination

The redox reaction was studied under weak acidic aqueous condition to exclude the autoxidation of caffeic acid which is particularly active at pH ≥ 7.0 [28]. The systems were kept under stirring at room temperature for all the test time. Kinetic measurements were carried out at room temperature on systems containing 60 μM CAF and V(V) concentration ranging from 30 to 120 μM. The systems were prepared by mixing appropriate aliquots of the CAF and V(V) solutions, brought separately to pH 2.8, 3.6, 4.0, 5.0 and 6.0 by addition of HClO_4_ or NaOH. To determine V(V) and V(IV), aliquots of stock solutions, buffered at pH 4.0, were passed through a Chelex-100 resin (previously conditioned with sodium acetate buffer at pH 4.0) in 5 ml glass columns (4 ml of hydrated resin). In this way the V(IV), formed during the reaction, was removed from solutions while V(V) was collected in the eluate. After rinsing the resin with water (15 ml), vanadium (IV) was eluted completely with 1N HCl (three bed volume). V(IV) and V(V) were then quantified using a Varian atomic absorption spectrophotometer with a Varian graphite furnace tube atomizer. The detection limit was 4 μg/L. V(V) was also determined in the form of V(V)-Desferri-Ferrioxamine B complex, whose absorbance was measured at 455 nm using an Agilent Technology Cary 60 UV-Vis spectrophotometer.

### CAF and oxidation products determination

CAF and its oxidation products were determined by HPLC as previously described [21]. Briefly, a Dionex DX-300 system, equipped with an UV-Vis Merk Hitachi Diode Array detector, and an Alltech Alltima C18 5U column was used. A H_2_O-acetonitrile-acetic acid (77.5%-17.5%-5.0%) mixture, brought to pH 3.5, was employed as the eluent at a flow rate of 0.5mL min^-1^ at room temperature.

The mass of CAF and of its oxidation products were achieved through their separation in LC-MS using an Agilent Technologies (Palo Alto, CA, USA) 1100 series LC/MSD equipped with a diode-array detector (DAD). A chemstation HP A.10.02 was used for data analysis. The chromatographic separation was achieved using a Luna C18 (150mm×4.6 i.d., 3 μm, Phenomenex, USA). The photodiode array detector was coupled to a mass spectrometer (quadrupole analyzer) directly to the sprayer needle where ions were generated by electrospray ionization (ESI) in both positive and negative ionization modes. Nitrogen was used as nebulizing and drying gas and different fragmentorvoltages were applied. Full scan data acquisition was performed scanning from 100 to 500 *m*/*z* using a cycle time of 2 s with step size of 0.1 υ.

### V(IV)-PGA system

The sorption of V(IV) by (PGA) were carried out at pH 3.0, 4.0, 4.5, 5.0 and 6.0 by dipping the polysaccharidic matrix (25 mg), into 100 mL of solutions containing 12 μmol of V(IV) (120 μM). The amount of V(IV) sorbed was quantified by the difference between the initial concentration and that found at equilibrium. The FT-IR spectra of the V(IV)-PGA samples, centrifuged at 5000 rpm (5°C), washed several times with water, dehydrated and stored under vacuum, were recorded on KBr disks (2 mg of sample in 100 mg of KBr) with a Nicolet 380 spectrophotometer (Thermo Fisher Scientific).

### V(IV)-, V(V)-, PGA-, -CAF-triticale plants (x *Triticosecale* Wittm.) systems

In order to determine the biological relevance of the redox reaction between V(V) and CAF, in the presence and absence of PGA, triticale plants (x *Triticosecale* Wittm.) were grown in the following aqueous solutions at pH 6.0: i) 120 μM V(V); ii) 120 μM V(V) + 120 μM CAF; iii) 120 μM V(V) + 120 μM CAF + 0.25 mg/mL PGA (equivalent to 56 μmol of carboxylic groups). Control plants were grown in 1 mM CaCl_2_ solutions which was used as background electrolyte for all the hydroponic solutions tested. Additional control plants were grown in 120 μM V(IV) solutions. Triticale plants were selected for these assays since CAF has not any phytotoxic effects against this species and because it revealed quite sensitive to heavy metal stress [21].

The phytotoxicity of all the solutions tested was determined by assessing the root and shoot length of triticale plantlets after 7 days of growth in a controlled environment (22°C temperature, 65% relative humidity) under natural light (February-March 2015). Seeds of winter triticale (cv. Universal) were germinated in the dark at 22°C as previously described [21]. Briefly, seeds were germinated in cylindrical polystyrene containers (3.5 cm height, 3.5 cm diameter) covered by a stainless steel net and filled to the rim with 30 mL of the treatment solution. In such a way seeds and growing roots were directly (and constantly) in contact with the treatment solutions [21]. After germination, triticale seedlings were grown under natural light and root and shoot length were measured after 7 days and their total vanadium content was determined. During the experiments, the containers were covered with foil to avoid root contact with light and photo-chemical reactions.

Each treatment solution was tested on a total of 30 plantlets (6 seeds per container x 5 replicates) and three independent experiments were carried out. Plant measurement data are reported as mean values ± standard errors. One-way analysis of variance was used to compare mean values and when significant *P*-values were obtained (*P*<0.05) differences between individual means were compared with the Fisher’s least significant difference test (*P*<0.05).

## Results and Discussion

### Stoichiometry of the redox reaction

Because of the monomer-oligomer equilibria present in the V(V) solutions [5,29], in this study we employed V(V) concentrations equal to or less than 120 μM. At concentrations as low as 120 μM, a significant amount (~15%) of trimer (V_3_O_9_)^3^ˉ is present [30]. A pronounced change in the V(V) distribution occurs if the concentration is in the millimolar or higher range as hydrolysis take place and a number of poly-nuclear species form [29,31]. In our experimental conditions, the pervanadyl ion (VO_2_^+^) is the predominant species at pH < 3.0 while at higher pH, H_2_VO_4_ˉ and HVO_4_^2^ˉ are the dominant species.

**Fig 1:**
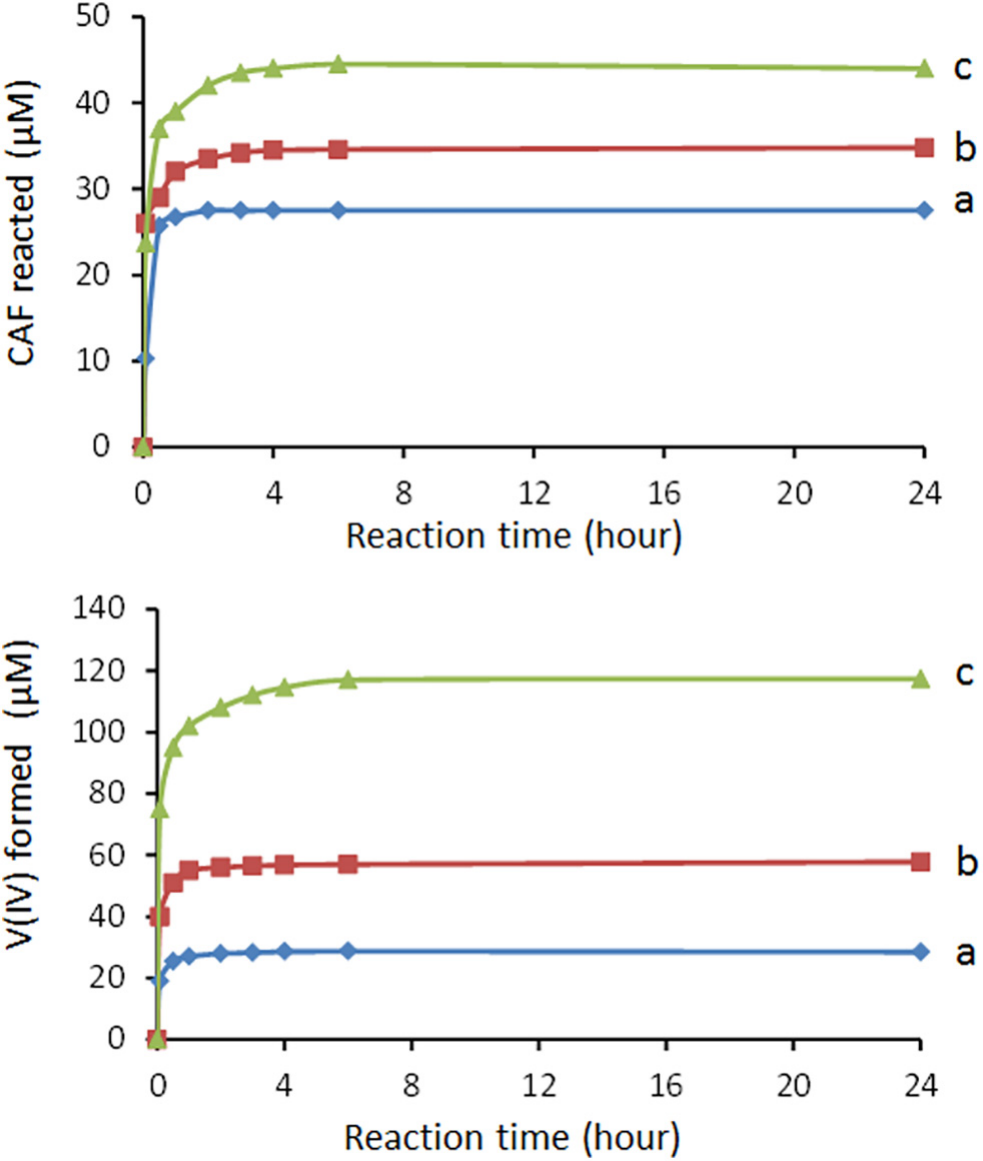
CAF reacted and V(IV) formed at pH 2.8 as a function of time and at different initial V(V) concentrations. Reaction conditions: CAF 60 μM; V(V) 30 μM (a), 60 μM (b) and 120 μM (c). Reaction volume 50 mL.

Tests on the redox activity of CAF towards V(V), carried out in the 2.8–6.0 pH range, evidenced a high ability of the biomolecule to reduce V(V) at pH 2.8. Thus, in order to determine the influence of the pH on the redox reaction, as a first step, we determined the stoichiometry of the reaction at pH 2.8. To this purpose, systems containing 60 μM CAF and 30, 60 and 120 μM V(V) (the V(V)/CAF molar ratio varied from 0.5 to 2.0) were studied. The amount of CAF oxidized, as a function of time and at different initial V(V) concentrations, is reported in Fig 1. The amount of V(IV) formed, at equilibrium, in the systems with a V(V)/CAF molar ratio equal to 0.5, 1.0 and 2.0 was equal to 28.5, 57.8 and 117.3 μM, respectively (Fig 1). The ratio between the amount of V(IV) produced (117.3 μM) and the CAF reacted (44.0 μM), relative to the system with V(V)/CAF molar ratio equal 2.0, indicates that the number of electrons involved in the reduction of the V(V) by one molecule of CAF is equal to 2.7, as it can be easily calculated from the ratio between the concentration in solution of VO^2+^ produced and CAF oxidized. Thus, the following stoichiometry at pH 2.8 can be proposed:

$$\text{CAF}+2.7\;{\text{VO}}_{2}{}^{+}+5.4\;{\text{H}}^{+}+2.7\;{\text{e}}^{-}\to 2.7\;{\text{VO}}^{2+}+\text{CAF oxidation products}+2.7\;{\text{H}}_{2}\text{O}$$

The proposed stoichiometry is in agreement with previous studies on the CAF electrochemical and/or chemical oxidation showing that the number of electrons released by CAF was larger than that expected by the oxidation of the OH phenolic groups to quinonic groups [32–34].

### Mechanism of the redox reaction

**Fig 2:**
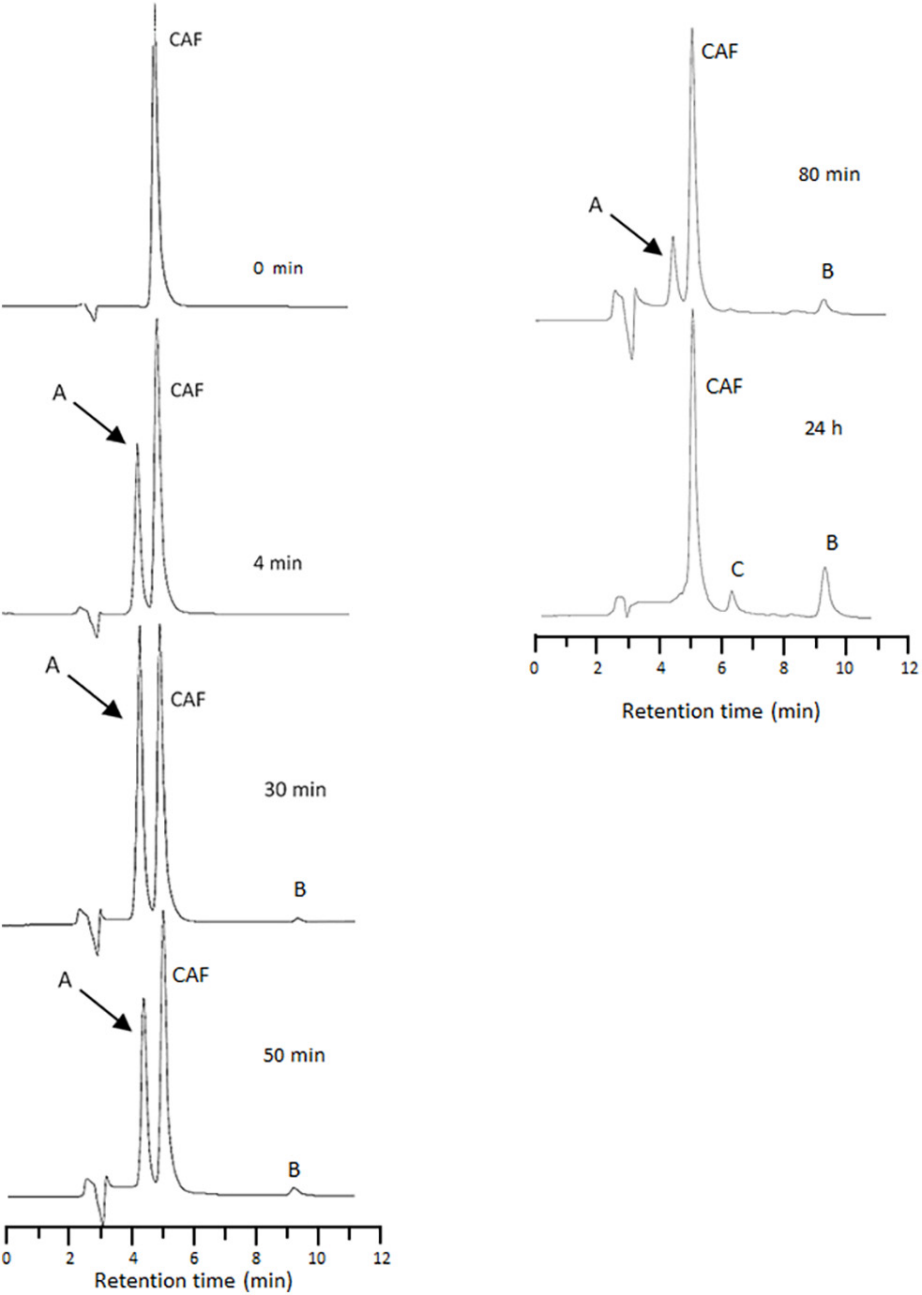
Chromatograms recorded at 254 nm and at different reaction times relative to the V(V)-CAF system at pH 2.8. V(V) 120 μM, CAF 60 μM.

**Fig 3:**
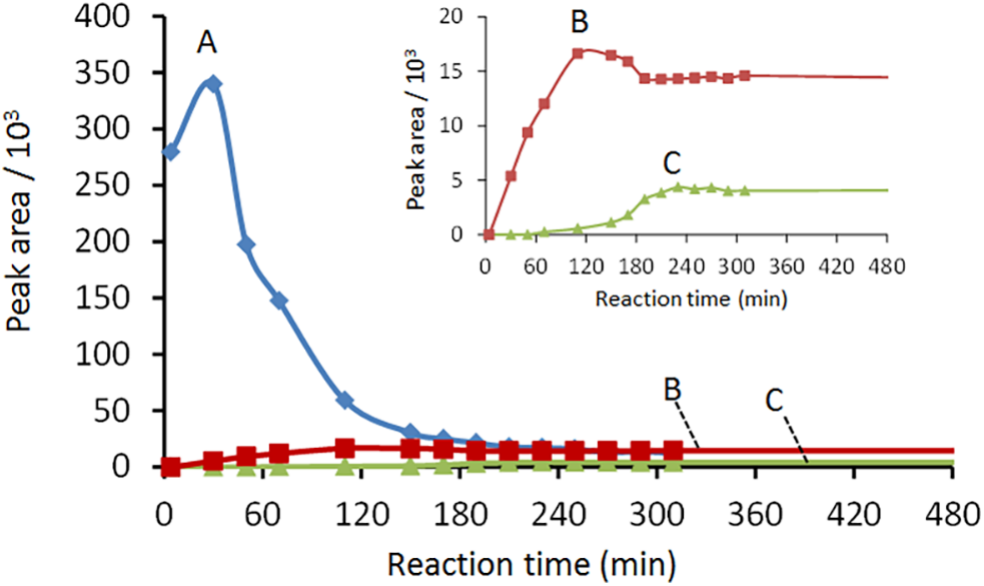
Peak area vs. reaction time curves for products A, B and C. Peak areas were obtained from HPLC-UV-DAD chromatograms (absorbance at 254 nm).

The HPLC tests show that CAF oxidizes more or less quickly depending on V(V) concentration leading to the formation of three main reaction products, with a retention time lower (product A) or higher (products B and C) than that of CAF. Fig 2 reports the chromatograms relative to the system with a V(V)/CAF molar ratio equal to 2, recorded at different reaction times, and at a wavelength of 254 nm. A similar trend was observed for the V(V)/CAF systems with a molar ratio equal to 0.5 and 1.0. The product A forms quickly at the beginning of the reaction and increases during the first 30 min, while decreases as the reaction proceeds (Fig 2). The decrease of the concentration of product A seems correlated with the appearance of product B, suggesting that A acts as a precursor of B while this latter seems to act as a precursor of product C. The trend of the different products as a function of time is reported in Fig 3.

**Fig 4:**
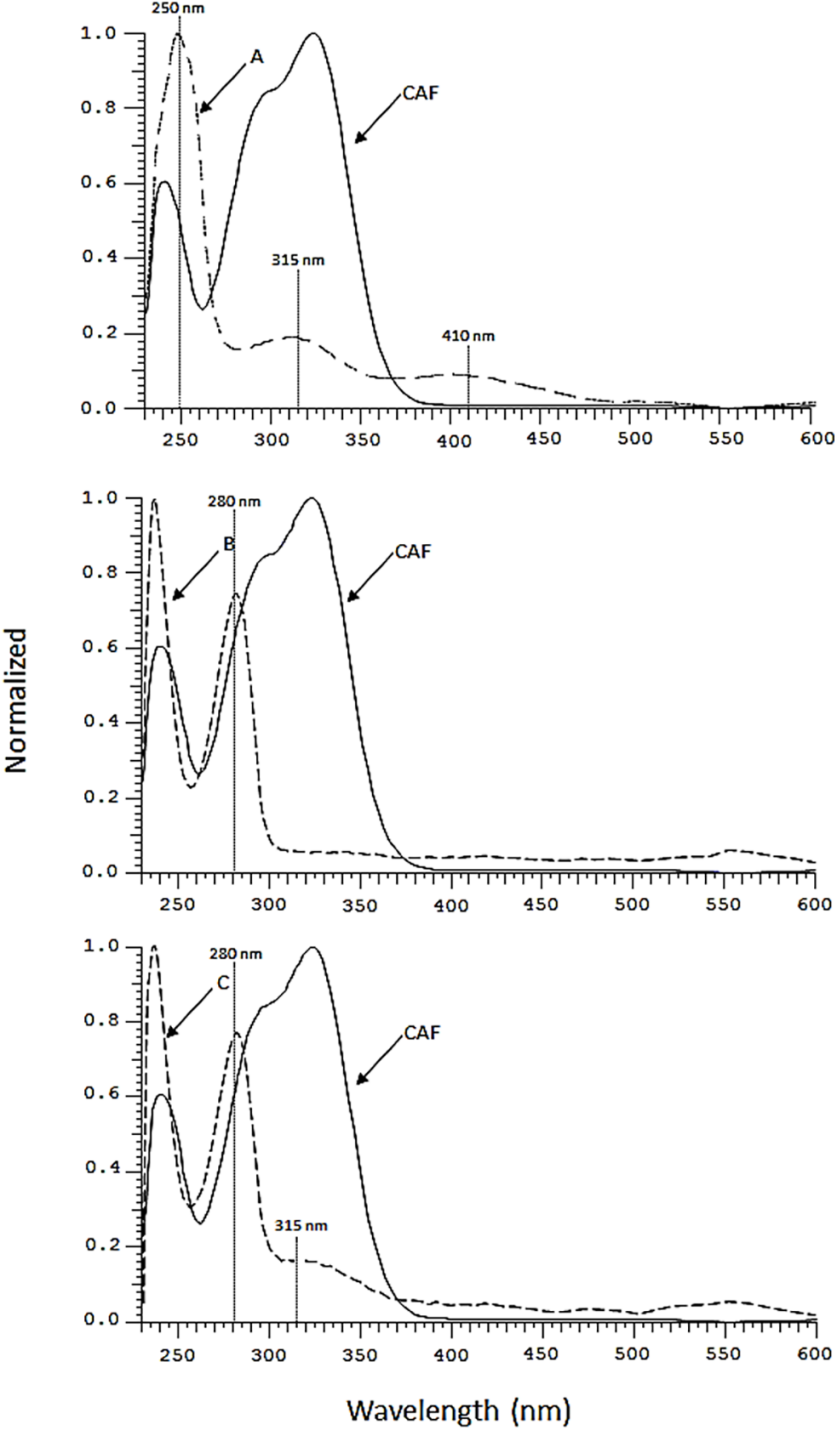
UV-Vis spectra of CAF (solid lines) and CAF-oxidation products (dashed lines, products A, B and C) recovered from the V(V)-CAF system at pH 2.8 after different reaction times, i.e. product A: 30 min, product B and C 24 h.

The A product exhibits an electronic absorption spectrum manly characterized by an absorption band at 250 nm and other two broads band located at 315 and 410 nm (Fig 4). This spectrum is very similar to that published previously [35] and attributed to CAF *o*-quinone formed following the CAF electrochemical and/or enzymatic oxidation. This suggests that the first step of the redox reaction is consistent with the formation of a CAF *o*-quinone. The UV-Vis spectrum of product B shows an intense absorption band at 280 nm while product C presents the same band at 280 nm accompanied by a shoulder band at 315 nm (Fig 4). The lack of the bands at 295 and 325 nm, which characterize the spectrum of CAF, indicates that these products are not CAF oligomers. Therefore, we can assume that products B and C can be formed by reactions involving the side chain of a CAF semiquinone molecule and one (or more) semiquinonic radicals to form dimers, trimmers, etc.

**Fig 5:**
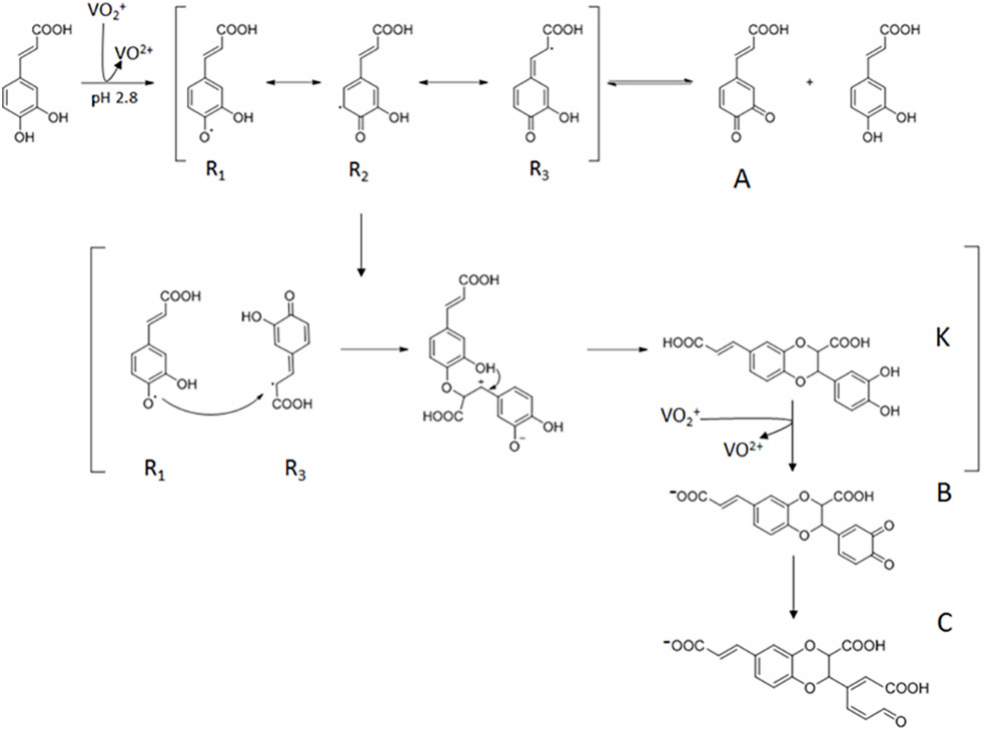
Likely mechanism of the redox reaction between CAF and V(V) at pH 2.8.

To assess this hypothesis, our samples were analyzed by mass spectrometry. The mass analysis indicates that products B and C, with molecular masses equal to 354 and 371 respectively, can be attributed to CAF dimers. The likely structures of products B and C, and their probable mechanism of formation, are reported in Fig 5. The first step of the reaction consists in an inner-sphere electron transfer from CAF to V(V) to give V(IV) and highly reactive phenoxy radicals (R_1_, R_2_, R_3_). Previous studies showed that the formation of *o*-quinone is a key step in its dimerization [36]. Continuous disproportionation of a pair of phenoxy radicals forms CAF and *o*-quinone. The reaction between CAF and *o*-quinone gave a pair of semiquinone radicals which, via random radical coupling, led to the formation of a dimer. The effect of the –CH = CH–COOH moiety in the electronic delocalization determines the energetic stability of the radical located on *ortho* position [37,38]. The product B (molecular ion: 354) likely derived from a K dimer (we were not able to detect its presence) originating by a coupling reaction (as an intermediate of one-electron oxidation) involving R_1_ and R_3_ radicals as reported in Fig 5. Two electrons are involved in the oxidation of the dimer K to provide the B product. The C compound (molecular ion: 371) could have originated by the breaking of the bond of the two adjacent quinonic groups of product B to give rise to the formation of a carboxylic and an aldehydic group. Such reaction can be promoted by the VO^2+^ ions following their interaction with the quinonic groups of product B.

The isolation of the B and C products to perform NMR and/or FT-IR studies to achieve a better structural characterization has proved very difficult so far, because of their transformation during storage, most likely promoted by the residual presence of vanadium ions as well as by the tendency of the dimmers to polymerize [39].

### Influence of the pH on the redox reaction

**Fig 6:**
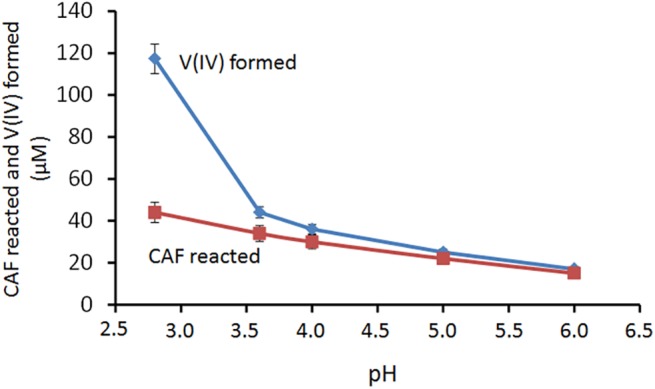
CAF reacted and V(IV) formed after 24 hours of reaction, as a function of pH, in the V(V)-CAF system with a V(V)/CAF molar ratio equal to 2.0. Initial conditions CAF 60 μM, V(V) 120 μM. Mean values ± standard deviations.

The redox reaction is strongly affected by the pH. Fig 6 reports, as a function of pH, the amount of CAF reacted and V(IV) formed after 24 hours of reaction, in the system with a V(V)/CAF molar ratio equal to 2.0.

The number of electrons released from a CAF molecule decreases as pH increases being about equal to 2.7, 1.3, 1.2, 1.1 and 1.1 at pH 2.8, 3.6, 4.0, 5.0 and 6.0, respectively.

The chromatographic analysis of the systems at pH 3.6 and 4.0 evidences the formation of the same products detected at pH 2.8 (A, B, and C), whereas at pH > 4.0 A and B are the only species detected.

The strong decrease in the redox activity may be justified by the fact that the standard redox potential (E°) for the V(V)-V(IV) couple [5] decreases with increasing pH, as it can be easily calculated by applying the Nerst equation. In particular, for the VO_2_^+^ + 2H^+^ + e^-^ ↔VO^2+^ + H_2_O (E° = 1.0 V) couple the redox potential is equal to 0.76 and 0.64 V at pH 2.0 and 3.0, respectively. The main species present at pH > 3.0 is the acid-base pair H_2_VO_4_ˉ ↔ HVO_4_^2^ˉ + H^+^ (pK_a_ = 8.1) being the H_2_VO_4_ˉ the predominant specie in the 3.5–6.0 pH range. By considering that the standard redox potential for the couple H_2_VO_4_ˉ + 4H^+^ + e^-^ ↔ VO^2+^ + 3H_2_O is 1.419 V, the redox potential at pH 4.0, 5.0 and 6.0 is equal to 0.47, 0.24 and 0.00 V, respectively. The half-peak potential (E_1/2_), relative to the couple CAF ↔ *o*-quinone + 2H^+^ + 2e^-^, was equal to 0.377, 0.362, 0.252, 0.162 V at pH 2.7, 3.0, 4.5 and 5.9, respectively [34].

As can be deduced by the trend in redox potentials, the redox reaction is favored in the 2.8–4.0 pH range, while is disadvantaged at pH values higher than 4.5. However, the redox activity recorded on the systems equilibrated at pH 5.0 and 5.8 can be due to the ability of CAF to bind V(IV) ions.

**Fig 7:**
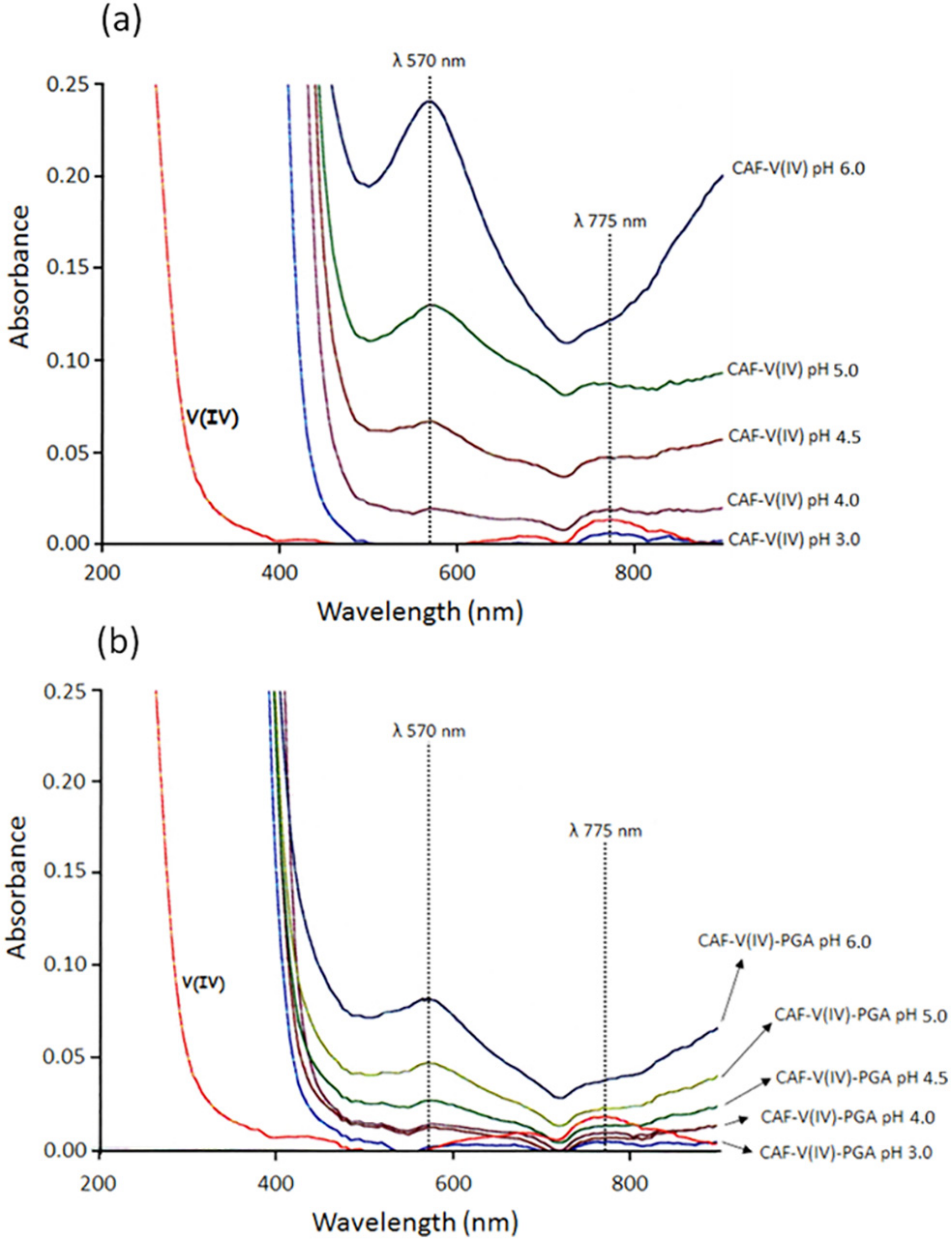
UV-Vis spectra of V(IV)-CAF (a) and V(IV)-CAF-PGA (b) systems at different pH values. CAF 1.2 mM, V(IV) 620 μM, PGA 0.25 mg/mL.

The UV-Vis spectra of solutions containing CAF/V(IV) in a 2:1 molar ratio, recorded in the 3.0–6.0 pH range, are characterized by the presence of two absorption bands located at 775 and 570 nm (Fig 7A). The sorption band at 775 nm is attributable to the VO(H_2_O)_5_^2+^ ion, while that at 570 nm is indicative of the involvement of carboxylic and/or catecholate groups of CAF in the metal coordination. The band at 775 nm disappears as pH increases and a concomitant increase of the band at 570 nm was observed. The band at 570 nm belongs to PhO^-^ → V (dπ) ligand-to-metal charge transfer (LMCT) transition [40,41]. Previous studies [42] report that V(IV) interact with caffeic acid leading to the formation of several complexes in which both the carboxylate and catechol or catecholate groups are involved. The ML is the main species in the 4.0–5.0 pH range. Such mode of binding (catecholic type) has been also suggested in the reaction between CAF and Cr(III) in weak acidic solution [43] and CAF and Al(III) ions [44].

### Redox activity of the V(V)-CAF-PGA system

**Fig 8:**
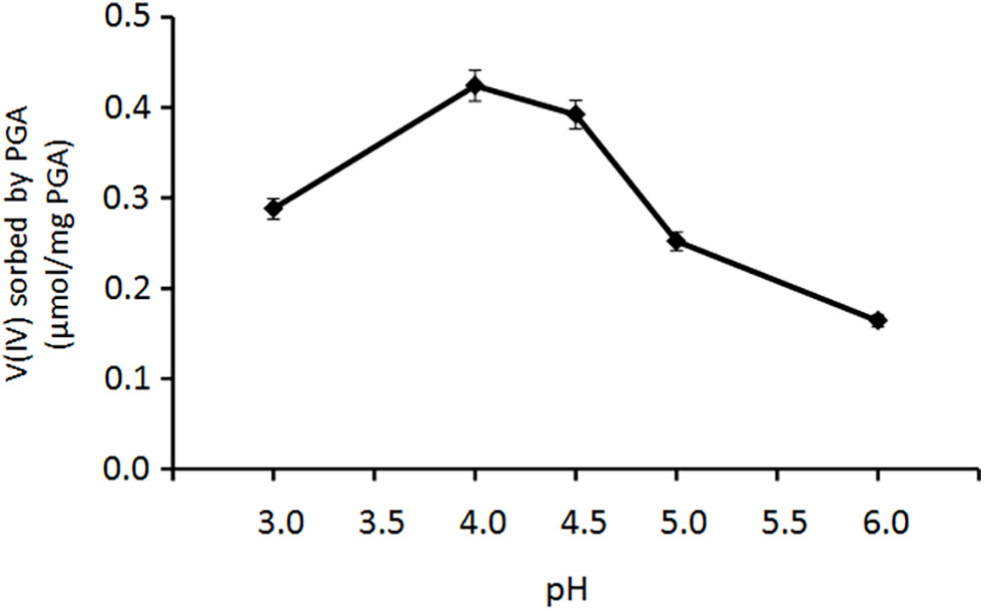
Sorption of V(IV) by 25 mg PGA at different pH values. Reaction volume 100 mL, V(IV) 120 μM. Mean values ± standard deviations.

In order to evaluate the role of the polygalacturonic acid (PGA) on the redox reaction, as a first step, we studied the interaction between V(IV) and PGA in the 3.0–6.0 pH range. Fig 8 reports, as a function of pH, the amount of V(IV) sorbed by 25 mg of PGA with an initial concentration of V(IV) equal to 120 μM. The amount of V(IV) sorbed increases as the pH increases from 3.0 to 4.0 and decreases from 4.5 to 6.0. This behaviour can be due to the VO^2+^ hydrolysis which leads to the formation of VOOH^+^ and (VOOH)_2_^2+^ species with lower affinity for the carboxylate groups of PGA.

**Fig 9:**
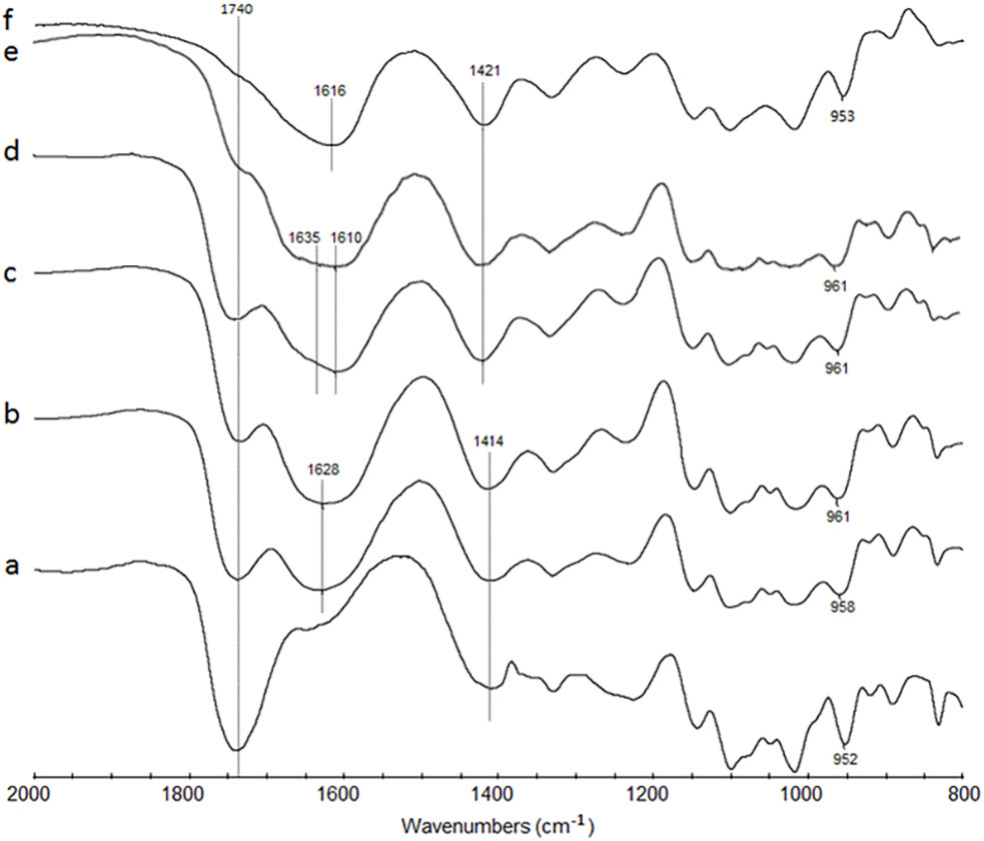
FT-IR spectra of HPGA (a), V(IV)-PGA systems at pH 3.0 (b), 4.0 (c), 5.0 (d) and 6.0 (e), and Na-PGA (f).

To verify such hypothesis, V(IV)-PGA samples were investigated by FT-IR spectroscopy. Fig 9 reports the FT-IR spectra of the V(IV)-PGA systems, at different pH values, polygalacturonic acid (HPGA) and Na-PGA.

The FT-IR spectra of the (HPGA) (Fig 9; spectrum a) is characterized by an absorption band at 1740 cm^-1^, assigned to the *ν*_(C = O)_ stretching frequency of the carboxylic group. This band progressively decreases following the replacement of the carboxylic hydrogen ion, as pH increases, and two strong bands became visible in the 1610–1635 and 1414–1421 cm^-1^ regions (Fig 9; spectra b-e). These latter bands can be assigned to the asymmetric (*ν*_as_) and symmetric (*ν*_s_) stretching mode of the carboxylate group, respectively. The magnitude of the difference between the *ν*_as_ and *ν*_s_ stretching (Δ*ν*) gives information about the type of interaction of the carboxylate groups with the metal ion [45,46]. The Δ*ν* value of the V(IV)-PGA system in the pH range 3.0–4.0 (Δν = 214; Fig 9, spectra b-c) is higher than that recorded for Na(I)-PGA (Δν = 195; Fig 9, spectrum f). This would suggest that carboxylate groups are directly involved in the coordination of the metal ion. As pH increases from 4.0 to 6.0, the band at 1628 cm^-1^ splitted into two new bands at 1635 and 1610 cm^-1^ (Fig 9; spectra d-e) suggesting that two V(IV)-PGA complexes coexist. In addition to the Δν = 214 cm^-1^ found for the systems equilibrated at pH 3.0 and 4.0, a Δν = 189 is found for the V(IV)-PGA samples equilibrated at pH 5.0 and 6.0. A similar value was found for Na-polygalacturonates (Fig 9; spectrum f) suggesting that also an electrostatic interaction regulates the V(IV) sorption by PGA. This can be due to the fact that at pH > 4.0, the V(IV) in solution is present manly as hydrolysed monuclear (VOOH^+^) and polynuclear ((VOOH)_2_^2+^) species, where the positive charge is lower or more delocalized than for VO^2+^ [30]. The band at 1018 cm^-1^, which characterizes the HPGA and Na-PGA systems, is not shifted to lower frequencies in the presence of VO^2+^ as found for Cu(II)-and Pb-PGA systems by [27] and [47] respectively. This suggests that the -OH groups and the oxygen ring of PGA are not involved in the coordination of the metallic ion to form multidentate complexes. The band at about 960 cm^-1^ (Fig 9; spectra b-e) can be assigned to the V = O stretching. This value is lower than that of VOSO_4_ 5H_2_O which is usually around 975–985 cm^-1^ [48]. The shift of the V = O bond to lower frequencies is indicative of a σ electron donation of the polysaccharidic matrix to antibonding orbitals of the V = O group.

**Fig 10:**
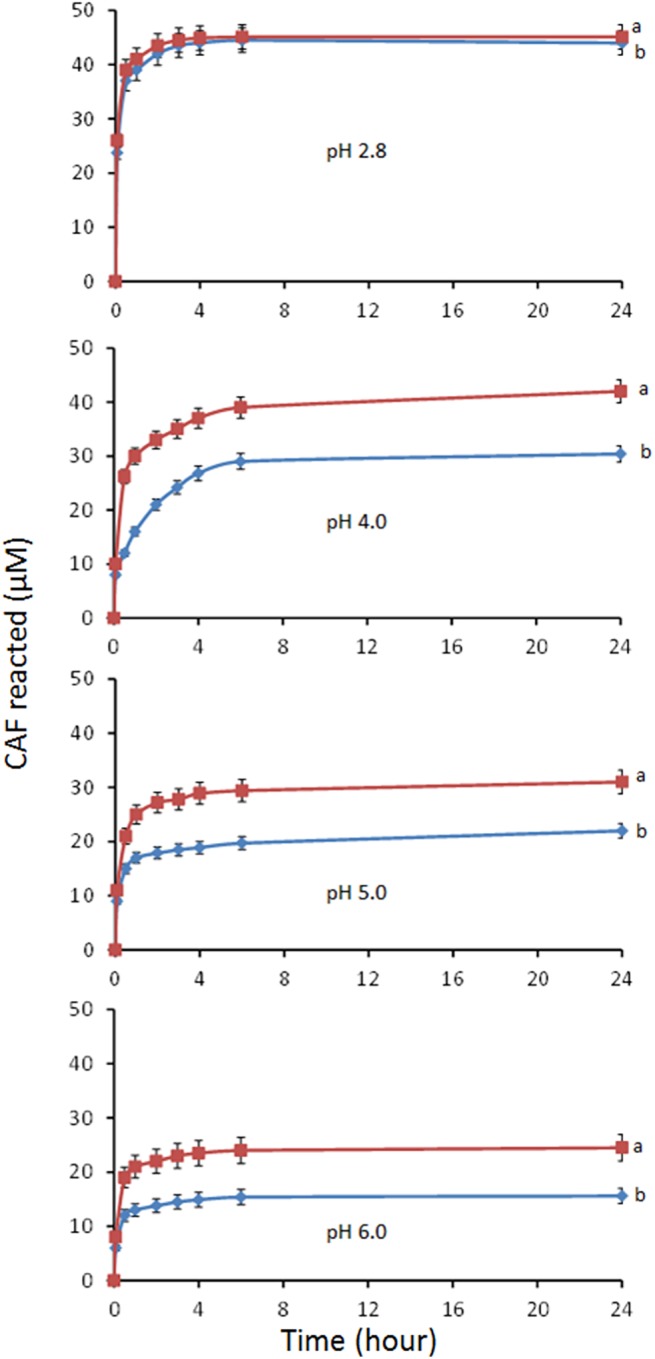
CAF reacted as a function of time in the V(V)-CAF-PGA (a) and V(V)-CAF (b) systems at different pH values. Reaction conditions: CAF 60 μM; V(V) 120 μM; PGA 0.25 mg/mL. Mean values ± standard deviations.

When PGA was added to the V(V)-CAF system, caffeic acid revealed more reactive particularly at pH 4.0, 5.0 and 6.0. This can be easily appreciated looking at the amounts of CAF oxidized by vanadate in 24 h in the presence and absence of PGA (Fig 10). Moreover, the concentration of the V(IV) formed at pH 2.8, 4.0, 5.0 and 6.0, in the V(V)-CAF-PGA systems was 0.6, 30, 36 and 58% higher compared to the respective binary V(V)-CAF systems. This behaviour can be explained by taking in account the higher affinity of the polysaccharidic matrix towards V(IV) with respect to CAF as can be deduced by the comparison of the UV-Vis spectra of the binary V(IV)-CAF and ternary V(IV)-CAF-PGA systems recorded in the 3.0–6.0 pH range (Fig 7A and 7B). The ratio between the absorbance values recorded at 570 nm, in the absence and presence of PGA, was equal to 2.8 in the whole pH range studied. This is consistent with Islam et al. [49] which highlighted how chelating ligands which stabilize a metal complex in a lower oxidation state can promote the reduction of the metal with a higher oxidation state. Similarly, [50] showed that NADPH reduces V(V) to V(IV) in the absence of enzyme catalysis when EDTA is present in the reaction mixture. In contrast, NADPH can only partially reduce V(V) in the absence of EDTA.

This result can be of great importance from a biochemical point view as pectic substances, acting as complexing agent towards V(IV), can cooperate with reducing agents to buffer the vanadium toxicity at the soil root-interface where pectic polymers and reducing agents are abundant. For instance, PGA could avoid the reaction of the reduced metal with hydrogen peroxide, which is readily converted by a Fenton-type reaction into hydroxyl radicals which can induce a multitude of effects on many cellular functions such as apoptosis [51].

### Biological relevance of the redox reaction between V(V) and CAF in the presence and absence of PGA

**Fig 11:**
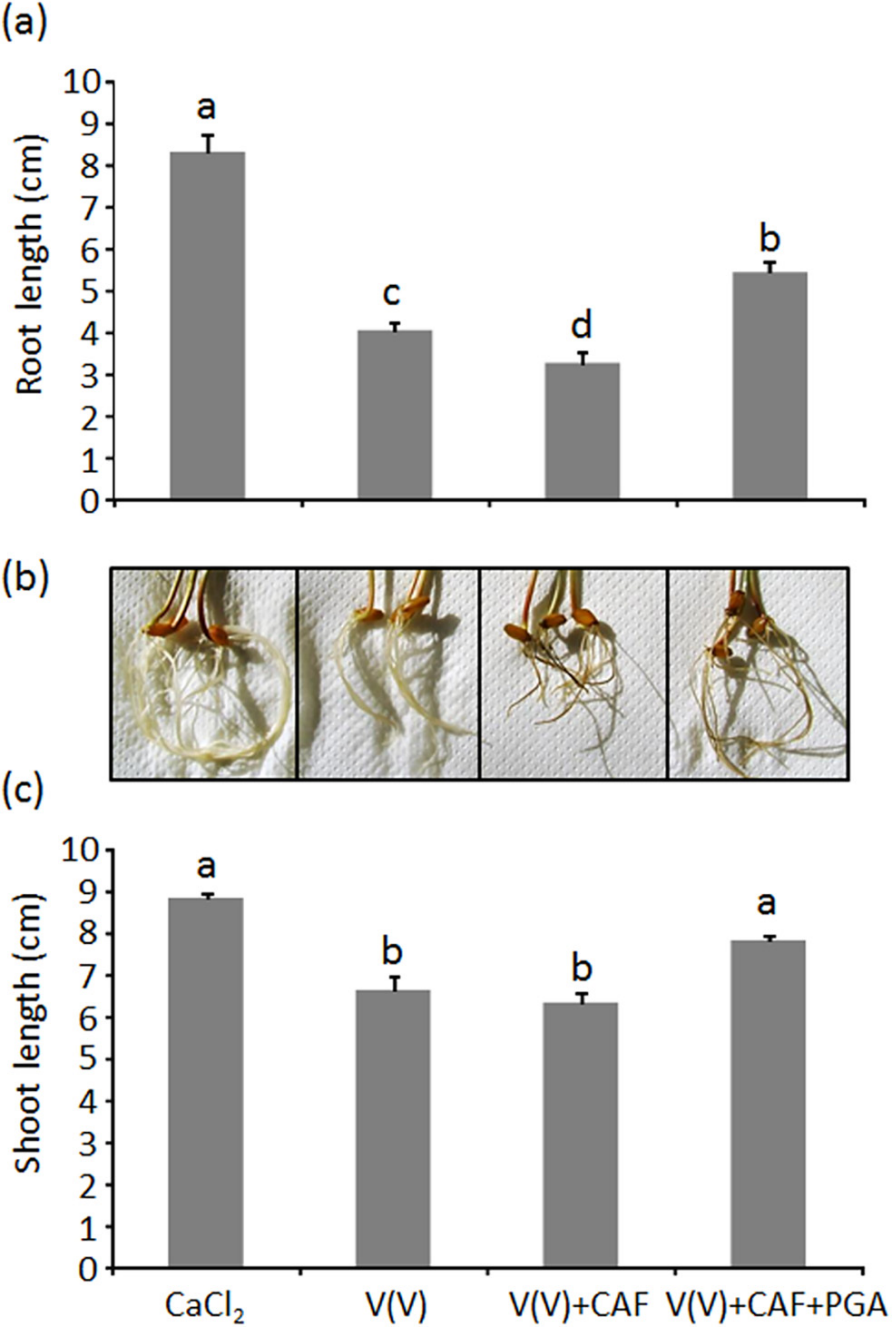
Root length (a), root appearance (b) and shoot length (c) of triticale plants grown for 7 days in 1 mM CaCl_2_ (control), 120 μM V(V), 120 μM V(V)+120 μM CAF and 120 μM V(V)+120 μM CAF+0.25 mg/mL PGA. CaCl_2_ (1 mM) was the supporting electrolyte for all the solutions tested. Means ± standard errors which share the same letter do not differ significantly according to the Fisher’s least significant difference test (LSD, *P*<0.05).

To evaluate the biological significance of the redox reaction between CAF and V(V), as well as the role of PGA in such reaction, triticale plants (x *Triticosecale* Wittm.) were grown at pH 6.0 in solutions containing V(V), V(V)+CAF and V(V)+CAF+PGA. The presence of 120 μM V(V) in the solution induced significant phytotoxicity effects on triticale plants. In particular, after 7 days of growth, root and shoot length was significantly reduced with respect to control plants (Fig 11A and 11C). This is consistent with the results reported by [52] which recorded a significant reduction of root length and shoot dry matter in cuphea plants (family Lythraceae) when V(V) was added to hydroponic solutions in the 25–150 μM range. Similar phytotoxic effects were also recorded for the V(V)-CAF system (Fig 11A and 11C). Since it was recently demonstrated that CAF has no phytotoxic effects against triticale [21], the observed results can be attributed to the presence in solution of V(V) and V(IV), this latter species originating from the redox reaction between CAF and V(V). The presence of V(IV), in the V(V)-CAF system, was also supported by the visual observation of typical symptoms induced by V(IV) such as dark roots (not observed in the V(V) system; Fig 11B) [11]. Such symptoms were apparent when triticale plants were grown in the presence of V(IV) (S1 Fig) but were also present in the V(V)-CAF-PGA system which, however, showed the least phytotoxicity (Fig 11A–11C). Root elongation was reduced by 35, 51 and 60%, with respect to control plants, for V(V)-CAF-PGA, V(V) and V(V)-CAF system respectively. On the other hand, shoot length in the V(V)-CAF-PGA system was not significantly different from that of control plants while it was approximately reduced by 26% in V(V) and V(V)-CAF systems (Fig 11 and S2 Fig). The lowest phytotoxicity recorded in the V(V)-CAF-PGA system can be explained by the ability of PGA to bind the V(IV) which originates from the redox reaction between CAF and V(V) (Figs 7–9), which ultimately leads to a lower concentration of V(V) in solution. The presence of PGA induced a significant reduction in the total vanadium accumulated in roots (33 μg/g d.m.) and shoots (3.7 μg/g d.m.) compared to plants grown in the V(V) and V(V)-CAF systems (44.5 and 5.5 μg/g d.m. for roots and shoots respectively, average values). Moreover, these latter concentrations were approximately 6-fold higher than those recorded in control plants.

These results highlight significant phytotoxic effects of the V(V)-CAF system at pH 6.0 which were comparable with those induced by V(V) alone. In the presence of PGA, however, the phytotoxicity of the V(V)-CAF system was significantly reduced confirming the high affinity of the polysaccharidic matrix towards the V(IV) formed and highlighting the *in vivo* relevance of PGA in the detoxification processes of V(IV) occurring at the soil-root interface.

## Conclusions

The results reported show that CAF can promote the reduction of V(V), highly toxic for living systems (e.g. V(V) acts as a phosphate competitor), to V(IV). Polygalacturonates, beside promoting the redox reaction, also act as effective buffering agents against the phytotoxicity of the V(IV) formed following CAF oxidation. On the basis of the results obtained we can retain that pectic substances, well represented in the apoplasm and at the soil-root interface, have an important role in the processes that regulate the reduction of V(V), not only by CAF, but also by other phenolic compounds and biological reducing agents such as NAD(P)H, ascorbate or cysteine.

## Supporting Information

S1 Fig Root appearance of triticale plants grown in the presence of 120 μM V(V) (a) and 120 μM V(IV) (b). CaCl_2_ (1 mM) was the supporting electrolyte in both solutions. Note typical dark roots of plants grown in the presence of V(IV).
(TIF)

S2 Fig Shoot biomass of representative triticale plants grown for 7 days in 1 mM CaCl_2_ (control), 120 μM V(V), 120 μM V(V)+120 μM CAF and 120 μM V(V)+120 μM CAF+0.25 mg/mL PGA. (TIF)
