# Supplementary figures and images for: Detoxification Processes from Vanadate at the Root Apoplasm Activated by Caffeic and Polygalacturonic Acids
Source: PLoS One. 2015 Oct 20;10(10):e0141041. doi: 10.1371/journal.pone.0141041 (PMC4618281; doi:10.1371/journal.pone.0141041)

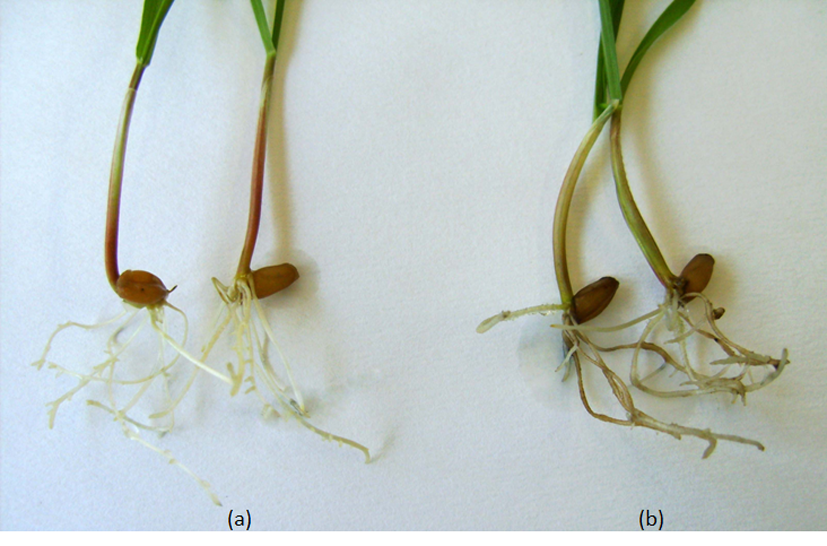

Supplement: S1 Fig — CaCl2 (1 mM) was the supporting electrolyte in both solutions. Note typical dark roots of plants grown in the presence of V(IV). (TIF) [file pone.0141041.s001.tif]

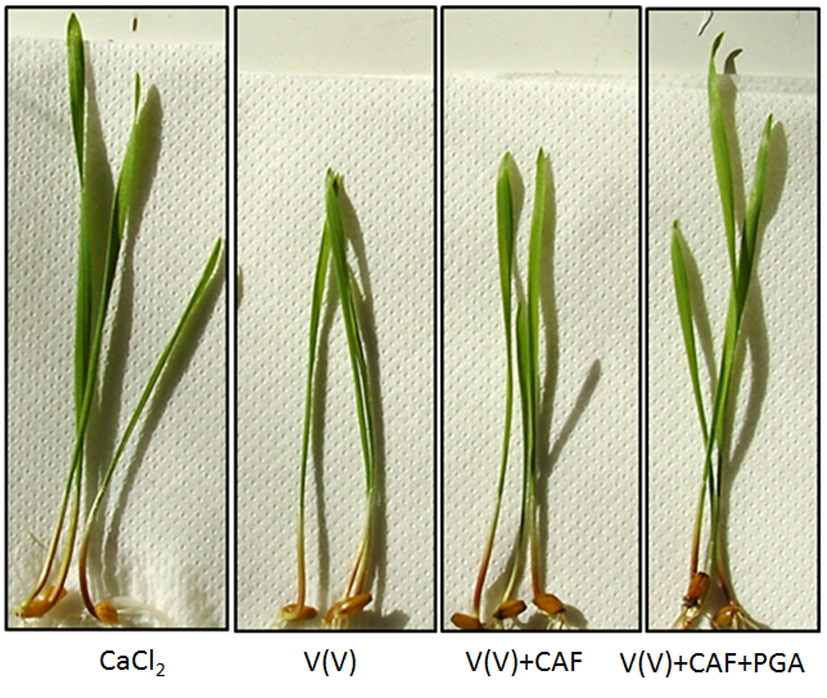

Supplement: S2 Fig — (TIF) [file pone.0141041.s002.tif]
